# Single vs. multiple fraction regimens for palliative radiotherapy treatment of multiple myeloma: A prospective randomised study

**DOI:** 10.1007/s00066-017-1154-5

**Published:** 2017-06-01

**Authors:** Milda Rudzianskiene, Arturas Inciura, Rolandas Gerbutavicius, Viktoras Rudzianskas, Andrius Macas, Renata Simoliuniene, Ruta Dambrauskiene, Greta Emilia Kiavialaitis, Elona Juozaityte

**Affiliations:** 1 Oncology Institute, Lithuanian University of Health Sciences, Eiveniu 2, 50009 Kaunas, Lithuania; 2 Anaesthesiology Department, Lithuanian University of Health Sciences, Kaunas, Lithuania; 3 Department of Physics, Mathematics and Biophysics, Lithuanian University of Health Sciences, Kaunas, Lithuania; 4 Intitute of Anesthesiology, University Hospital Zurich, Zurich, Switzerland

**Keywords:** Osteoclastic bone loss, Survival, Pain relief, Recalcification, Quality of life, Osteoklastischer Knochenverlust, Überleben, Schmerzlinderung, Rekalzifizierung, Lebensqualität

## Abstract

**Purpose:**

To compare the impact of a single fraction (8 Gy × 1 fraction) and multifraction (3 Gy × 10 fractions) radiotherapy regimens on pain relief, recalcification and the quality of life (QoL) in patients with bone destructions due to multiple myeloma (MM).

**Patients and methods:**

In all, 101 patients were included in a randomised prospective clinical trial: 58 patients were included in the control arm (3 Gy × 10 fractions) and 43 patients into the experimental arm (8 Gy × 1 fraction). The response rate was defined according to the International Consensus on Palliative Radiotherapy criteria. Recalcification was evaluated with radiographs. QoL questionnaires were completed before and 4 weeks after treatment.

**Results:**

Pain relief was obtained in 81/101 patients (80.2%): complete response in 56 (69%) and partial in 25 patients (30.9%). No significant differences were observed in analgesic response between the groups. Significant factors for pain relief were female gender, age under 65, IgG MM type, presence of recalcification at the irradiated site. Recalcification was found in 32/101 patients (33.7%): complete in 17 (53.2%) and partial in 15 (46.2%). No significant differences were observed in recalcification between the groups. Significant factors for recalcification were Karnofsky index ≥ 60%, haemoglobin level ≤ 80 g/dl, MM stage II and analgesic response at the irradiated site. The QoL after radiotherapy was improved in the control group.

**Conclusion:**

The same analgesic and recalcification response was observed using two different radiotherapy regimens. Higher doses should be used to achieve a better QoL.

## Zusammenfassung

**Zielsetzung:**

Vergleich der einzeitigen vs. fraktionierten palliativen Radiotherapie in Bezug auf Schmerzlinderung, Knochenrekalzifizierung und Lebensqualität (QoL) bei Patienten mit multiplem Myelom (MM).

**Patienten und Methoden:**

In die randomisierte, prospektive Studie wurden 101 Patienten eingeschlossen: Die Kontrollgruppe (*n* = 58) erhielt eine fraktionierte (3 Gy × 10 Fraktionen) und die Experimentgruppe (*n* = 43) eine einzeitige Radiotherapie (8 Gy × 1 Fraktion). Ossäre Läsionen wurden radiologisch nach den Kriterien des Internationalen Consensus der palliativen Radiotherapie evaluiert. Die Rekalzifizierung wurde mittels Röntgenaufnahmen ermittelt. QoL-Fragebögen wurden vor Beginn und 4 Wochen nach Behandlung beantwortet.

**Ergebnisse:**

Insgesamt 81/101 Patienten (80,2 %) zeigten eine Schmerzreduktion: vollständiges bei 56 (69 %) und partielles Ansprechen bei 25 Patienten (30,9 %). Zwischen den untersuchten Gruppen ergab sich kein signifikanter Unterschied bezüglich der Schmerzreduktion. Wesentliche Faktoren für die Schmerzlinderung waren weibliches Geschlecht, Alter < 65 Jahre, IgG-MM-Typ sowie bereits vorhandene Rekalzifizierung der osteolytischen Läsionen. Eine Rekalzifizierung zeigte sich bei 32/101 Patienten (33,7 %): vollständig in 17 (53,2 %) und partiell in 15 Patienten (46,2 %). Zwischen den Gruppen zeigte sich kein signifikanter Unterschied bei der Rekalzifizierung. Einflussnehmende Faktoren für die Rekalzifizierung waren ein Karnofsky-Index ≥ 60 %, ein Hämoglobingehalt ≤ 80 g/dl, ein MM-Stadium II und vorhandene Analgesie an der bestrahlten Stelle. Nach Radiotherapie stieg die QoL nur in der Kontrollgruppe.

**Schlussfolgerung:**

Zwischen den beiden Strahlentherapieregimen zeigte sich kein signifikanter Unterschied bei der Schmerzbesserung und der Rekalzifizierung, jedoch besserte sich die QoL nur nach multiplen Fraktionen signifikant.

## Introduction

Skeletal related events is one of the signs of multiple myeloma (MM) [1, 2]. Osteoclastic destructions reduce patients’ quality of life (QoL) and decreases patient survival [3].

Bone pain is the first sign of MM for 70% of patients and the patients receive radiation at least once during their MM therapy [4]. Where radiotherapy is applied, pain can be reduced by 75–100% [4–11]. Recalcification of bone destruction is observed in 40–60% [4, 6, 11, 12].

Results of previous clinical trials have shown the same effect of pain relief and recalcification when applying different radiotherapy regimens for treatment of patients with solid tumour metastases [13–16]. This data, however, cannot be directly applied in treatment of patients with MM, since their future prospects are better [4]. The medical literature provides only a small number of studies evaluating various radiotherapy regimens for treatment of patients with MM [4–12]. No randomized prospective study has been carried out worldwide to date comparing multifraction and single fraction regimens for treatment of patients with MM bone disease and the impact on pain relief, recalcification and QoL. The aim of this prospective study was to evaluate these endpoints raising the hypothesis that one single fraction has the same analgesic and recalcification effect as compared to multifraction therapy.

## Patients and methods

From 2010–2015 a randomized prospective clinical trial was performed at the Lithuanian University of Health Sciences. Multifraction radiotherapy regimen (3 Gy × 10 fractions) was applied to the control group of patients and single fraction regimen (8 Gy × 1 fraction) was applied to the experimental group. In all, 58 patients were included in the control arm and 43 patients were included in the experimental arm. A random sampling was performed by a computerised programme. Inclusion criteria were the following: age over 18 years, diagnosis of MM according to the International Myeloma Working Group’s Criteria [17], presence of painful bone destructions or impending fracture verified by radiographs, Karnofsky index (KI) above 40%, written informed consent. Exclusion criteria were the following: presence of bone metastases from solid tumours, solitary plasmacytoma, prior irradiation at the same site, inability to complete the QoL questionnaires, patients that could not be monitored. The study protocol was prepared in accordance with the Helsinki Declaration and was approved by the Lithuanian Regional Research Ethics Committee. Informed consent was obtained from all the participants prior to enrolment in the study.

A total of 101 patients (65 women and 36 men, median age: 66.6 years, range 43–88 years) were included in the study. Patients’ characteristics are detailed in Table 1.

**Table 1:** Patients’ characteristics

Characteristics	Control group, No. (%)	Experimental group, No. (%)	*p*-value
*Gender*			
Male Female	20 (34.5) 38 (65.5)	16 (37.2) 27 (62.8)	0.777^a^
*Age (years)*			
Mean (SD) ≤ 65 years > 65 years	66.60 (10.42) 25 (43.1) 33 (56.9)	68.72 (7.99) 10 (23.3) 33 (76.7)	0.251^c^ 0.038^a^
*Karnofsky index (%)*			
Median (range; mean)	60 (50–80; 59.14)	60 (50–80; 61.63)	0.152^d^
*Radiotherapy for patients with:*			
Newly diagnosed MM Prior MM history	26 (44.8) 32 (55.2)	14 (32.6) 29 (67.4)	0.21 ^a^
*Clinical stage (Durie Salmon)*			
II III	11 (19) 47 (81)	5 (11.6) 38 (88.4)	0.318 ^a^
*Paraprotein*			
IgG IgA Light chains IgM Nonsecretory	38 (65.5) 9 (15.5) 10 (17.2) 0 1 (1.8)	31 (72.1) 2 (4.7) 9 (20.9) 1 (2.3) 0	0.217^b^
*Irradiated sites*			
Spinal vertebrae Pelvic bone Extremities	41 (70.7) 12 (20.7) 5 (8.6)	18 (41.9) 16 (37.2) 9 (20.9)	0.013^a^
*Surgery*			
Yes No	10 (17.2) 48 (82.8)	11 (25.6) 32 (74.4)	0.307^a^
*Bisphosphonates*			
Yes No	11 (19) 47 (81)	8 (18.6) 35 (81.4)	0.963^a^
*Concurrent chemotherapy*			
High-dose dexamethasone Other chemotherapy: Bortezomib-based chemotherapy Immunomodulator-based chemotherapy None	35 (60.3) 12 (20.7) 7 (12.1) 6 (10.3) 11 (19)	27 (62.8) 10 (23.3) 8 (18.6) 2 (4.7) 6 (13.9)	0.792^a^
*Pain score at admission*			
0–4 5–7 8–10	11 (18.9) 15 (25.9) 32 (55.2)	4 (9.3) 15 (34.9) 24 (55.8)	0.328^a^
*Pain medication*			
Opioid Non-opioid	45 (77.6) 11 (18.9)	34 (79.1) 5 (11.6)	0.382 ^a^
*Opioid dose (mg/day)*			
Median (range; mean)	60 (10–260; 73.44)	60 (10–210; 68.12)	0.627^d^

*SE* standard error of mean, *SD* standard deviation, *MM* multiple myeloma

^a^χ^2^ test

^b^Fisher’s exact test

^c^Student’s t test for independent populations,

^d^Mann–Whitney U test

Pain intensity was assessed according to the visual analogue scale (VAS) [18]. A pain score ≤ 4 was classified as mild, 5–7 as moderate and ≥8 as severe [19]. Analgesics were divided: opioid and non-opioid. A dose of opioid analgesics was converted to a mean morphine-equivalent dose (MED; in mg/day) [20]. Pain intensity and the dose of analgesics was evaluated before radiotherapy and after 4, 12 and 24 weeks. Recalcification was independently measured by two radiologists comparing radiographs before radiotherapy and after 4 and 12 weeks. An initial assessment of the radiologists’ comparisons was performed prior to study initiation and found no difference.

QoL was assessed by using EORTC QLQ-C30 version 3 and EORTC QLQ-MY20 QoL questionnaires [21, 22]. The patients’ responses of single items were linearly transformed from 0–100 scores according to the EORTC scoring rules [23]. High points in the functional scales and in the global health status scale indicate a good functional status, whereas high points in the symptom scales indicate a poor status of health. QoL was evaluated before radiotherapy and 4 weeks post treatment.

The analgesic response rate was defined according to the International Consensus on Palliative Radiotherapy criteria [24].

Since there is no common criteria of recalcification, we used criteria from other studies [6, 25]: complete response was defined as full reossification of treated osteolysis, while partial response was defined as evidence of marginal osteosclerosis around the lesion without complete reossification.

Acute toxicity was assessed in the first 4 weeks after radiotherapy by applying RTOG (Radiation Therapy Oncology Group)/EORTC (European Organisation for Research and Treatment of Cancer) toxicity criteria [26].

Statistical data analysis was performed by using the IBM SSPS Statistics 23 for Windows (SPSS Inc., Chicago, IL, USA). The χ^2^ test and Fisher’s exact test for small expected frequencies were used to compare proportions among groups created by sociodemographic and clinical characteristics. McNemar test was used to compare the proportions of pain type before and after treatment. Wilcoxon signed-rank test was used to compare values of quantitative features not distributed by Normal law between two related populations. The Mann–Whitney U test was used to compare values of quantitative features not distributed by Normal law between two independent groups and Kruskal–Wallis test was used to compare them among three or more independent groups. Results of the analysis are presented as median (mean score and range: minimum–maximum value). Means of quantitative data distributed by Normal law between two independent populations were compared using Student’s t‑test for independent populations. Observed differences were accepted as statistically significant if *p*-value < 0.05. Influence of demographic, clinical and symptom variables to pain relief, recalcification and QoL was analysed using binary logistic regression method. Stepwise variable removal procedure (Backward conditional) was used to determine a model with variables which influence is statistically significant: all analysed parameters were entered to the initial logistic model and at each step of the procedure the least significant parameter was removed from the model until all remaining parameters showed a statistically significant influence on pain relief, recalcification or QoL. Findings of the model with the biggest Nagelkerke pseudo coefficient of determination which indicates goodness of fit of the model, and with the biggest percent of correct classification of all cases are published in the article. Results of the analysis are presented as odds ratio (OR) and 95% confidence interval (95% CI) of odds ratio. The influence of demographic, clinical and symptom variables to pain relief, recalcification and QoL was considered as statistically significant if the confidence interval of odds ratio did not include the value 1.

## Results

### Pain relief

**Fig. 1:**
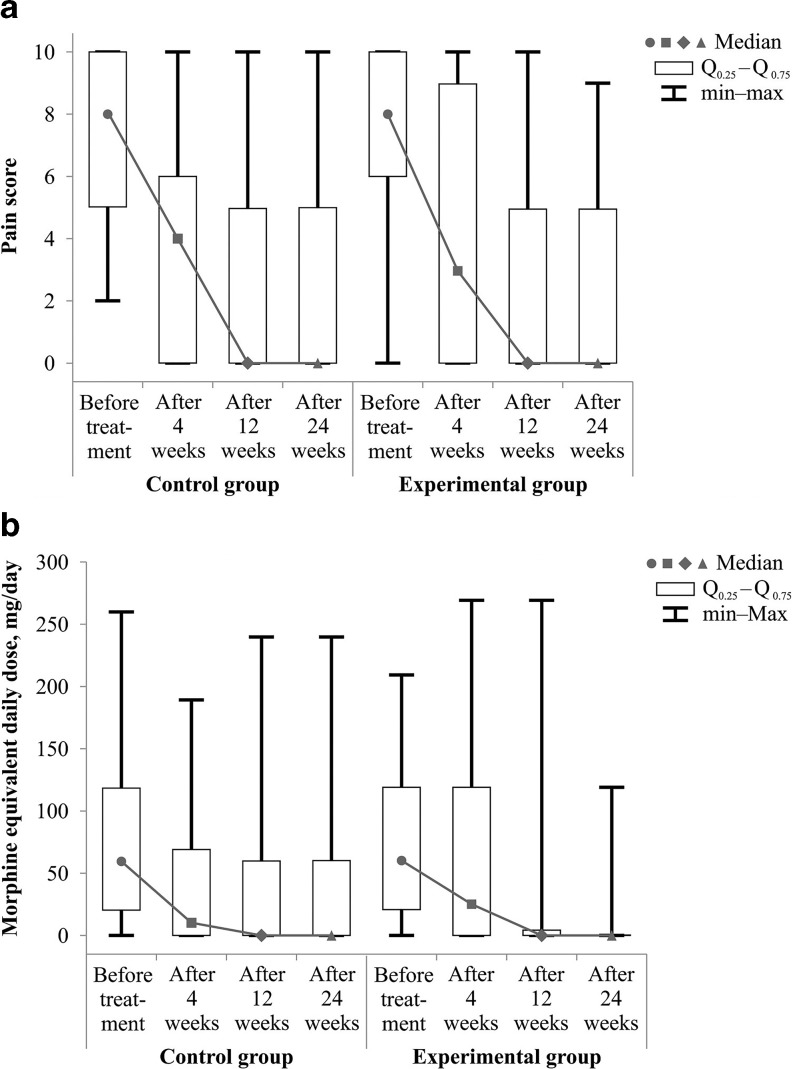
Patient self-reported pain score (**a**) and use of opioid analgesics (**b**) in the control and experimental groups before treatment and during the follow-up period

All patients had been suffering from pain prior to radiotherapy. The pain was mild in 15 patients (59%), moderate in 30 (29.7%) and severe in 56 (55.4%). Thirty-six patients (64.3%) who indicated severe pain before treatment felt significantly less pain during 4 weeks after radiotherapy (McNemar *p* < 0.001). Patients in the control group before treatment reported a median VAS of 8 (range 2–10, mean 7.4), 4 weeks after radiotherapy their median VAS was 4 (range 0–10, mean 3.6), after 12 and 24 weeks the median VAS was 0. Patients in the experimental group before treatment reported a median VAS of 8 (range 2–10, mean 7.5), 4 weeks after radiotherapy the median VAS was 3 (range 0–10, mean 4.2), after 12 and 24 weeks the median VAS was 0. No significant differences were observed in the groups in the median of pain score before therapy nor in its decrease during the monitored period (Fig. 1).

Sixteen patients (15.8%) were using non-opioid drugs prior to radiotherapy and all of them ceased analgesic intake during the first 4 weeks after treatment. Seventy-nine patients (78.2%) were taking opioid analgesics. Consumption of opioid analgesics was significantly reduced at 4 weeks after radiotherapy (Wilcoxon *p* = 0.001). The median of the MED used before the treatment in the control group was 60 (mean score 73.4, range 10–260) and in the experimental group it was also 60 (mean score 68.1, range 10–210). Four weeks after radiotherapy, in the control group the median MED was 10 (mean score 44.2, range 0–190) and in the experimental group MED was 25 (mean score 58.7, range 0–270). At 12 and 24 weeks after radiation treatment, the median MED was 0 in both groups. No significant differences were observed in the groups in the median MED before treatment nor in its decrease during the monitored period (Fig. 1).

During the follow-up period pain relief was obtained in 81 patients (80.2%): complete response in 56 (69%) and partial response in 25 (30.9%). Manifestation of analgesic response is demonstrated in Table 2. No significant differences were observed between the groups. The treatment arms were not balanced for age or sites of irradiation.

**Table 2:** Analgesic response after radiation treatment

	Control group, *n* (%)	Experimental group, *n* (%)	*p* value
Overall response	49 (84.5)	32 (74.4)	0.209
Complete response	34 (69.4)	22 (68.8)	0.952
Partial response	15 (30.6)	10 (31.2)	

Manifestation of analgesic response in the patient groups was tested by applying χ^2^ criterion, *p* < 0.05

Univariate statistical analysis revealed that the age under 65 years (*p* = 0.016), disease stage II (*p* = 0.03) and recalcification in the irradiated site (*p* = 0.011) were significant parameters for analgesic response, whereas other parameters (gender, KI, paraprotein type, haemoglobin level, surgery, pain score at admission, total radiation dose, bisphosphonates, concurrent chemotherapy) were not statistically significant.

All parameters mentioned above were included in the binary logistic regression model for analysis of their influence on pain relief: female gender, age under 65 years, IgG MM type, presence of recalcification in the irradiated site have a significant impact on analgesic response. Other factors analysed were not statistically significant (Table 3).

**Table 3:** Significant factors to analgesic response after radiotherapy in binary logistic analysis

Parameter		OR (95% CI)	*p* value
Gender	Female vs male^a^	9.0 (1.01–80.53)	*0.049*
Age (years)	<65 vs ≥65^a^	10.99 (1.15–105.03)	*0.037*
Paraprotein	IgG vs other type^a^	16.41 (1.85–145.85)	*0.012*
Recalcification in the irradiated site	Presence vs absence^a^	15.99 (1.27–200.76)	*0.032*

Significant parameters are in italic. Entire sample was analyzed

^a^Reference group

### Recalcification

Bone X‑ray images of 95 patients (94.1%) were evaluated for recalcification, X‑ray images of 6 patients were excluded due to early death. Recalcification was found in 32 patients (33.7%): complete in 17 (53.2%) and partial in 15 (46.2%). Manifestation of recalcification is demonstrated in Table 4. No significant differences were observed between the groups.

**Table 4:** Manifestation of recalcification response after radiation treatment

	Control group, *n* (%)	Experimental group, *n* (%)	*p* value
Overall response	18 (32.1)	14 (35.9)	0.703
Complete response	7 (38.9)	10 (71.4)	0.067
Partial response	11 (61.1)	4 (28.6)	
Stable destruction	31 (55.4)	17 (43.6)	0.259
Progressing destruction	7 (12.5)	8 (20.5)	0.292

Manifestation of recalcification response in the patient groups was tested by applying χ^2^ criterion, *p* < 0.05

Univariate statistical analysis revealed that KI ≥ 60% (*p* = 0.004) and pain relief in the irradiated site (*p* = 0.011) were significant parameters for recalcification, whereas other parameters were not statistically significant.

All the parameters mentioned above were included in the binary logistic regression model for analysis of their influence on recalcification: KI ≥ 60%, haemoglobin level ≤ 80 g/dl, II stage of MM and analgesic response in the irradiated site have a significant impact on recalcification. Other analysed factors were not statistically significant (Table 5).

**Table 5:** Factors significant to recalcification in binary logistic analysis

Parameter		OR (95% CI)	*p* value
Karnofsky index (%)	≥60% vs <60%^a^	3.93 (1.22–12.65)	*0.022*
Haemoglobin level (g/l)	≤80 vs >80^a^	2.72 (1.57–13.02)	*0.01*
Clinical stage (Durie–Salmon)	II vs III^a^	2.73 (1.81–9.23)	*0.023*
Pain perception after radiation treatment	Decrease vs no decrease^a^	5.54 (1.15–26.55)	*0.032*

Significant parameters are in italic. Entire sample was analysed

^a^Reference group

### Quality of life

All the patients completed questionnaires before and after radiotherapy. Respondents completed the questionnaires independently.

Univariate statistical analysis revealed that KI ≥ 60% (*p* = 0.004), radiotherapy to pelvic bones (*p* = 0.038) and mild pain at admission (*p* = 0.004) were significant parameters for better evaluation of QLQ-C30 global health status scale before radiotherapy.

In the control group comparison of QLQ-C30 global health status, symptom, functional scales and QLQ-MY20 symptom scales revealed significant improvement of QoL after radiotherapy (*p* = 0.004, *p* = 0.003, *p* = 0.017 and *p* = 0.034 respectively). Interestingly, the QoL after radiotherapy was only significantly improved in the control group (Table 6).

**Table 6:** Evaluation of QLQ-C30 and QLQ-MY20 before and after radiation therapy. Significant parameters are in italic

	Control group		*p* value	Experimental group		*p* value
	Before RT	After RT		Before RT	After RT	
QLQ-C30 global health scale median (min–max; mean)	*16.7 (0–83.3; 23.3)*	*16.7 (0–83.3; 32.3)*	*0.004*	16.7 (0–75; 26.9)	16.7 (0–75; 28.3)	0.606
QLQ-C30 symptom scales median (min–max; mean)	*33.3 (6.8–87.7; 39.4)*	*24.4 (14.2–81.5; 35.1)*	*0.003*	50 (18.5–92.6; 45.9)	39.5 (23.5–92.6; 48.1)	0.181
QLQ-C30 functional scales median (min–max; mean)	*75.5 (10–133; 83.9)*	*87.3 (9–133; 88.4)*	*0.017*	49.3 (0–133; 60.2)	50.3 (0–133; 62.2)	0.854
QLQ-MY20 symptom scales median (min–max; mean)	*33.3 (15–80; 39.3)*	*33.3 (7.2–76.7; 36.8)*	*0.034*	41.7 (15–95; 49.9)	47.2 (24.4–98.3; 50.7)	0.94
QLQ-MY20 functional scales median (min–max; mean)	66.7 (0–133.3; 84.9)	77.8 (0–133; 87.4)	0.3	61.1 (0–133.3; 62.8)	61.1 (0–133; 63.1)	0.987

Wilcoxon signed-rank test, *p* < 0.05

### Side effects

Acute toxicity was evaluated in the first 4 weeks after radiotherapy. The side effects were uncommon, low grade and reversible. No significant difference was found between the groups.

## Discussion

### Pain relief

Radiotherapy produces an analgesic effect by inhibiting chemical pain mediators and causing tumour shrinkage. The effect of radiation dose on pain relief is a matter of debate. The results of randomized clinical studies of palliative radiotherapy of bone metastases from solid tumours do not show superiority of any particular radiotherapy regimen [13–16, 27, 28]. The role of different radiotherapy regimens for MM is not well established [4–12].

Some studies did not find a significant difference between the dose of radiation and pain reduction [4, 7, 9, 11]; however, Adamietz et al. [5] and Minova et al. [10] reported the need for higher doses to obtain adequate pain relief. The current study confirms the efficacy of 8 Gy single fraction radiotherapy: the overall analgesic response was 74%, most patients achieved pain relief in the first 12 weeks and analgesic effect remained throughout the follow-up period. Binary logistic regression did not show a significant impact of dose on pain relief.

In studies reported by Adamietz et al. [5] and Mose et al. [11] concurrent chemotherapy had a significant impact on a positive response to radiotherapy, but our and other studies did not show this relationship [4, 9]. Lack of correlation with chemotherapy may be because chemotherapy effectively reduces tumour bulk but its effect on local symptoms is not always sufficient.

Mose et al. [11] reported that the high KI had an impact on a positive analgesic response. The opposite was found in the study performed by Stolting et al. [4]. This corresponds with our experience.

### Recalcification

According to the literature recalcification occurs in 40–50% of the irradiated bone destructions [4, 6, 11, 12]. The effect of radiation dose on recalcification is a matter of debate.

Koswig and Budach [29] found that multifraction regimens significantly increase the bone density in the area of metastases compared with single fraction; also Stolting et al. reported that recalcification was detected at total doses >40 Gy for MM patients [4]. Balducci et al. [6] found recalcification with median total doses of 38 Gy. However, the study published by Mose et al. [11] and our experience did not show any influence of radiation dose on recalcification.

Stolting et al. [4] reported the importance of concurrent chemotherapy for recalcification. Mose et al. [11] found that chemotherapy reinforces stabilization of the irradiated bone. In our study we did not find any impact of chemotherapy on recalcification. This could be due to the fact that chemotherapy reduces tumour bulk but there is little data for bone remodelling in patients treated with proteasome inhibitors. In our study only 14.9% of patients received bortezomib; therefore due to the small sample we cannot draw any conclusion on its impact on recalcification.

Mose et al. [11] reported that the high KI and receipt of bisphosphonates had an impact on recalcification. Also we found that a KI > 60% has a positive impact on recalcification. The use of bisphosphonates was insignificant but this may be due to the small sample of patients (only 18%) who were using bisphosphonates.

### Quality of life

Novel therapies have led to an improvement in survival, which has resulted in an increase in symptom burden due to the disease itself and the effects of treatments [30, 31]. There are some clinical trials that analyse the effect of radiotherapy on QoL in the treatment of patients with metastases, but there is no clinical study in the treatment of patients with MM. The Dutch Bone Metastasis Study did not show differences in QoL between the single and multifraction regimens [32]. Some studies reported that patients who have pain relief after radiotherapy also have a better QoL [33–35]; however, Sauer et al. [36] considered that radiotherapy leads to pain relief, but QoL is not affected positively due to side effects.

Caissie et al. [34] did not find a correlation between the improvement in QoL and the total radiation dose. We found that patients in the control group experienced significant improvement in QoL after radiotherapy. This could be associated with the fact that there were younger patients and a higher total equivalent dose was prescribed, which could lead to better disease control and improvement in QoL. We evaluated QoL before and 4 weeks after radiotherapy and a longer follow-up period evaluating QoL might have shown an even greater improvement. Thus, more studies are needed to address this observation in more detail.

Two studies showed that higher KI correlate with better QLQ-C30 scales [37, 38]. This corresponds with our data. In contrast to Cassie et al. [38], we found that radiotherapy to pelvic bones was a significant parameter for better evaluation of the QLQ-C30 global health status scale.

This study has potential limitations. The treatment arms were imbalance by age and the irradiated sites which could be a reason that QoL was improved in the control group. Additionally the logistic regression showed that age under 65 years has significant impact on pain relief. In the control group there were more young patients; thus this age discrepancy should be taken into consideration when comparing pain relief between groups.

## Conclusion

Our study revealed no significant differences in the analgesic and recalcification response between two different radiotherapy regimens; however, only multiple fraction radiotherapy achieved a significant improvement in QoL. Our study also suggests multiple fractionation regimens if a better QoL is important.

## References

[1] Raab MS, Podar K, Breitkreutz I (2009). Multiple myeloma. Lancet.

[2] Raje N, Roodman GD (2011). Advances in the biology and treatment of bone disease in mutiple myeloma. Clin Cancer Res.

[3] Terpos E, Morgan G, Dimopoulos MA (2013). International Myeloma Working Group recommendations for the treatment of multiple myeloma – related bone disease. J Clin Oncol.

[4] Stolting T, Knauerhase H, Klautke G (2008). Total and single doses influence the effectiveness of radiotherapy in palliative treatment of plasmocytoma. Strahlenther Oncol.

[5] Adamietz IA, Schober C, Schulte RW (1991). Palliative radiotherapy in plasma cell myeloma. Radiother Oncol.

[6] Balducci M, Chiesa S, Manfrida S (2011). Impact of radiotherapy on pain relief and recalcification in plasma cell neoplasms: long-term experience. Strahlenther Onkol.

[7] Bosch A, Frias Z (1988). Radiotherapy in the treatment of the multiple myeloma. Int J Radiat Oncol Biol Phys.

[8] Yaneva MP, Goranova-Marinova V, Goranov S (2006). Palliative radiotherapy in patients with multiple myeloma. J BUON.

[9] Leigh BR, Kurtts TA, Curtis FM (1993). Radiation therapy for the palliation of multiple myeloma. Int J Radiat Oncol Biol Phys.

[10] Minowa Y, Sasai K, Ishigaki T (1996). Palliative radiation therapy for multiple myeloma. Nippon Igaku Hoshasen Gakkai Zasshi.

[11] Mose S, Pfitzner D, Rahn A (2000). Role of radiotherapy in the treatment of multiple myeloma. Strahlenther Oncol.

[12] Manfrida S, Chiesa S, Rossi E (2010). Impact of radiotherapy on pain relief and recalcification in patients affected by plasma cell neoplasms: a long term experience. J Clin Oncol.

[13] Chow E, Harris K, Fan G (2007). Palliative radiotherapy trials for bone metastases: a systematic review. J Clin Oncol.

[14] Foro Arnalot P, Fontanals AV, Galcerán JC (2008). Randomized clinical trial with two palliative radiotherapy regimens in painful bone metastases: 30 Gy in 10 fractions compared with 8 Gy in single fraction. Radiother Oncol.

[15] Sande TA, Ruenes R, Lund JA (2009). Long-term follow-up of cancer patients receiving radiotherapy for bone metastases: results from a randomised multicentre trial. Radiother Oncol.

[16] Bone Pain Trial Working Party. (1999). 8 Gy single fraction radiotherapy for the treatment of metastatic skeletal pain: randomized comparison with a multifraction schedule over 12 months of patient follow-up. Bone Pain Trial Working Party. Radiother Oncol.

[17] International Myeloma Working Group (2003). Criteria for the classification of monoclonal gammopathies multiple myeloma and related disorders: a report of the International Myeloma Working Group. Br J Haematol.

[18] Jensen MP, Karoly P, Braver S (1986). The measurement of clinical pain intensity: a comparison of six methods. Pain.

[19] Chow E, Doyle M, Li K (2006). Mild, moderate or severe pain categorized by patients with cancer with bone metastases. J Pall Med.

[20] Selby & York Palliative Care Team & Pharmacy Group (2011). Palliative care analgesic dose conversion chart. 03/2006 Review date 01/2011.

[21] Aaronson NK, Ahmedzai S, Bergman B (1993). The European Organization for Research and Treatment of Cancer QLQ – C30: a quality-of-life instrument for use in international clinical trials in oncology. J Natl Cancer Inst.

[22] Stead ML, Brown JM, Velikova G (1999). Development of an EORTC questionnaire module to be used in health-related quality-of-life assessment for patients with multiple myeloma. Br J Haematol.

[23] Fayers P, Aaronson NK, Bjordal K, Curran D, Bottomley A (2001). EORTC QLQ – C30 scoring manual.

[24] Chow E, Wu JS, Hoskin P (2002). International consensus on palliative radiotherapy endpoints for future clinical trials in bone metastases. Radiother Oncol.

[25] Harada H, Katagiri H, Kamata M (2010). Radiological response and clinical outcome in patients with femoral bone metastases after radiotherapy. J Radiat Res.

[26] Cox JD, Stetz J, Pajak TF (1995). Toxicity criteria of the Radiation Therapy Oncology Group (RTOG) and the European Organization for Research and Treatment of Cancer (EORTC). Int J Radiat Oncol Biol Phys.

[27] Sze WM, Shelley MD, Held I (2003). Palliation of metastatic bone pain: single fraction versus multifraction radiotherapy – a systematic review of randomized trials. Clin Oncol.

[28] Wu JS, Wong R, Johnston M (2003). Meta-analysis of dose-fractionation radiotherapy trials for the palliation of painful bone metastases. Int J Radiat Oncol Biol Phys.

[29] Koswig S, Budach V (1999). Remineralization and pain relief in bone metastases after after different radiotherapy fractions (10 times 3 Gy vs. 1 time 8 Gy). A prospective study. Strahlenther Onkol.

[30] Cömert M, Güneş AE, Sahin F (2013). Quality of life and supportive care in multiple myeloma. Turk J Haematol.

[31] Mols F, Oerlemans S, Vos AH (2012). Health-related quality of life and disease-specific complaints among multiple myeloma patients up to 10 yr after diagnosis: results from a population-based study using the PROFILES registry. Eur J Haematol.

[32] Steenland E, Leer JW, van Houwelingen H (1999). The effect of a single fraction compared to multiple fractions on painful bone metastases: a global analysis of the Dutch Bone Metastasis Study. Radiother Oncol.

[33] Zeng L, Chow E, Bedard G (2012). Quality of life after palliative radiation therapy for patients with painful bone metastases: results of an international study validating the EORTC QLQ – BM22. Int J Radiat Oncol Biol Phys.

[34] Caissie A, Zeng L, Nguyen J (2012). Assessment of health-related quality of life with the European Organization for Research and Treatment of Cancer QLQ – C15-PAL after palliative radiotherapy of bone metastases. Clin Oncol (R Coll Radiol).

[35] Valesin Filho ES, de Abreu LC, Lima GH (2013). Pain and quality of life in patients undergoing radiotherapy for spinal metastatic disease treatment. Int Arch Med.

[36] Sauer N, Leising D, Wild B (2006). Pain and quality of life following palliative radiotherapy of bone metastases. Strahlenther Onkol.

[37] Lam K, Chow E, Zhang L (2013). Determinants of quality of life in advanced cancer patients with bone metastases undergoing palliative radiation treatment. Support Care Cancer.

[38] Caissie A, Culleton S, Nguyen J (2012). EORTC QLQ-C15-PAL quality of life scores in patients with advanced cancer referred for palliative radiotherapy. Support Care Cancer.

